# Human Health and Christianity in the Context of the Dilemma of Forgiveness

**DOI:** 10.1007/s10943-021-01424-1

**Published:** 2021-09-13

**Authors:** Jarosław Horowski, Mirosław Kowalski

**Affiliations:** 1 Nicolaus Copernicus University in Toruń, Toruń, Poland; 2 University of Zielona Góra, Zielona Góra, Poland

**Keywords:** Forgiveness, Disadvantages of imprudent forgiving, Communicating forgiveness, Forgiveness and health, Reasonable forgiveness

## Abstract

This article argues that Christianity has the potential to strengthen people’s health when solving the forgiveness dilemma. However—paradoxically—the starting point for the analysis is the presumption that a hasty and imprudent decision to forgive may negatively impact the health of the decision-maker, and that Christianity may contribute to people making unconsidered decisions by prompting them to forgive. In the first part of the analysis, the concept of health and its biblical understanding are discussed. The second part includes both a reflection on forgiveness-related dilemmas and the tension between the decision to forgive and the feeling of regret that may negatively influence health. In the third part, the Christian concept of forgiveness with reference to the aforementioned issues is discussed.

## Introduction

This analysis aims to evaluate the influence Christianity exercises on people’s health that both provides justifications for the decision to forgive and motivates such decisions. Although forgiveness is perceived as a process whose final result is the victim’s well-being (Crespo, 2007) and psychological studies indicate the importance of the decision to forgive for the well-being of the decision-maker (Harris & Thorsen, 2005; Purcell et al., 2018), the uncritical approach to forgiving raises doubts, especially if the decision results not from personal volition but is influenced by groups or the culture to which the person belongs. Similarly, the influence of religion, of Christianity in particular, on the decision about forgiveness and the related well-being of the individual, is affirmed and confirmed in psychological studies (Bassett et al., 2016; Breslin & Levis, 2008; Głaz, 2019; Lee & Kim, 2021; Randal & Bishop, 2013; Worthington et al.,^2016^; ^2019^; Worthington & Wade, 2020; Zarzycka, 2019). However, the interdependencies indicated in these empirical studies do not testify that each decision to forgive is good and positively affects the victim’s health (Grün, 2015).

The authors’ reflection on the issue takes on a philosophical character. This means that the article focuses on forgiveness not as a process but as a decision, leading to consequences for the social, psychological and spiritual dimensions.

The starting point for this analysis is the holistic concept of health, which is expressed through the inner harmony of the physical, mental and spiritual spheres. An essential condition for this harmony is bringing order to a person’s relationship with the surrounding reality, including interpersonal relationships (Antonovsky, 1979). These relationships are of central interest to this article. Accordingly, the authors recognise a twofold conditioning for individuals’ health: on the one hand, achieving harmony, especially between the feelings and decisions of individuals in the context of experiencing harm and, on the other hand, pursuing the aims set in relationships with people important to them, but who have caused the harm.

Just as maturing to forgive is a process (sometimes very lengthy), health and disease are understood as dynamic processes in which they interpenetrate each other (O’Toole, 2003). Due to the philosophical nature of the article, we assume that forgiveness is treated as a decision; that is, the issue of the aggrieved parties’ struggling with an experience that is difficult for them, including, for example, becoming aware of the harm and its consequences, seeking solutions other than forgiveness, and discovering their merits and defects, has been ignored. Since we focus on the decision to forgive itself (and not on the forgiveness process), this analysis can be understood as a reflection on the possible impact of forgiveness decisions on the forgiving parties’ state of health or illness. The discussion of the issue concerning the victims’ struggle with harm and the decision to forgive, having their own dynamics and influence on the individuals’ health, would require a separate article.

The article is divided into three parts, each covering two or three points. The first part presents the understanding of health, which is a reference point for the analysis of the addressed issue. The second part is devoted to the relationship between health and forgiveness, and the third part approaches the subject of forgiveness from a Christian perspective. Thus, a foundation is laid for the verification of the proposed thesis. The following question underlies these deliberations: Is it not somewhat peculiar that when so much importance is attached to empiricism and the value of “facts”, so little attention has been paid to theoretical analysis and empirical research on perhaps the most vital aspect of the Christian religion, that is, forgiveness, in terms of its influence on human health?

### Health as a Multidimensional Reality

Almost every person, when asked what the most important thing in their life is, will answer without hesitation that it is health. It is in health, or in being healthy, that we see the basis for happiness and success in our private and professional lives. Health is the existential fundament for defining plans and achieving goals. Simultaneously, health is variously understood and conceptualised (Gaweł, 2018; Kowalski & Gaweł, 2006). In this discussion, the authors accept several general assumptions about the understanding of health.

Firstly, health concerns four levels of human functioning: physical—pertaining to physical or somatic health; mental—mental health is traditionally measured by the degree of the integration of personality; social—social health refers to the relationship between the individual and the community (it is measured by the degree of syntony, that is, social concordance; the ability to maintain relationships with others and perform social roles); and spiritual—concerning spiritual health (Kowalski & Gaweł, 2006; Syrek, 2000).

Writing about health, Jean Watson (1989) claims that people as living creatures exist in three realms of nature, namely mind, body and soul. These three spheres form their Selves, their egos. Watson then proceeds to connect the notion of health with that of wholeness and harmony of the threefold human Self, pertaining to the harmony between “my Self” and the “Self of others” as well as between “my Self” and the surrounding nature. Consequently, it is reasonable to state that “healthy is not so much the organism as the human being—the person” (Wulff et al., 1991). Reducing health only to the physical dimension, as in the biomedical model, may prove to be dangerous for individuals. One may also point to the “persons in their health”/ “persons in their illnesses” approach that brings persons to the fore in both their life and everyday relationships.

Secondly, the concept of health is closely related to the individual and their non-transferable human experience, and it refers to the path of personal development as the foundation of human existence. Therefore, the concept should be analysed from the perspective of individuals’ personal nature and development as persons. Since health is also a basic condition for the realisation of human freedom and personal growth, it refers, firstly, to the person’s bodily sphere, and secondly, to their psycho-spiritual foundation. It is, therefore, necessary to distinguish between experiencing health and being a healthy person, that is, one who does not suffer from any ailment. Obviously, in the latter case, it is necessary to understand that ailments do not refer only to malaise or physical pain. At the same time, it is worth mentioning that “what is meant here is an ailment in the sense proper to a person, i.e. one that implies a straining of, if not outright discarding, their natural potentialities dictated by their spiritual-bodily nature” (Wróbel, 1999, p. 149).

Bearing the foregoing discussion in mind, it should be said that health is a state in which a person—in individual and social dimensions—is free from difficulties impeding self-realisation. Human health is the ability to exercise self-determination and the freedom to act to the highest degree, and thus to overcome situations that may weaken a person’s vitality and potential (Wróbel, 1999). Logically, illness is a state that interferes with who the persons are and with what is proper to them when they are unable to deal with a given situation and overcome it in everyday life, or when—driven by responsibility—they cannot remain indifferent to it. Hence, a defined state of illness is conditioned by the situational–personal level, and it is against this background that the causes of deterioration in a person’s health should be considered.

In the aforementioned context—which is also situated within the thematic scope of this article—it is worth recalling Carl Rogers’ proposal for understanding human health (and partially bodiliness). Rogers reconciles two contradictory explanatory perspectives: the biological (whereby the individual is understood solely through the prism of physiological activities) and the philosophical (in which the deficiencies of empirical premises are compensated for by axioms from axiology and philosophical anthropology). The focal feature of this project is the location of all conscious and vegetative phenomena in the holistically understood human body. Each individual has access to the body through experience. The pillar of Rogers’ theoretical framework is the category of self-actualisation and the assumption of the secondary character of human consciousness in relation to the world of values. People do not so much construct values in a substantive (ontological) sense, as they assign a valuable identity to the objects of their experience. In this way, persons create their own unique individuality—a subjectively conditioned and diversified axiological world, which in the case of a healthy person does not depend on external evaluative systems. Thus, the postulate of one’s own identity corresponds to identifying valuable qualities and classifying them within a gradation free of others’ prejudice. Identity is born based on health in both the empirical and purely theoretical sense. According to Rogers, health consists in the integration into a coherent whole of diverse experiences, achieving the harmony of individual structures of the Self and the biological tendencies of the organism. Individuals are healthy insofar as they can entirely rely on their senses. Only then, does a constructive developmental drive activate in them, based on their own potentialities (Rogers, 2002).

### A common Ground for Health and Forgiveness

Thirdly, health has a fundamental meaning for humans, and forgiveness has a specific purpose. These constructs theoretically and practically combine a range of meanings referring to different aspects of experience and cognition. Health—with regard to a person’s forgiveness—is related to the deed and the quality of relations that the person establishes. Forgiveness, on the other hand, as a direct individual act between persons, from the theological, sociological and psychological perspectives, binds persons in many ways. In the theological sense, one can point out that forgiveness, by heightening a person’s awareness of others, emphasises the value of health. On the other hand, forgiveness—in the context of human health—is a strategy for coping with life’s adversities; it is a source of contextual meaning and social interactions. Enabling control over experiences, forgiveness allows one to obtain a sense of control over situations, reduce anxiety and have a sense of development—empowering one to achieve a higher level of self-realisation. Since health (e.g. in the social dimension) concerns the relationship between the individual and the community (referring to individuals’ ability to maintain proper relations with other people and perform social roles), it should be noted that through the act of forgiveness, a person not only facilitates the expression of “individual strategies of emotion management”, i.e. of regulating and controlling emotions, but also helps adopt a coping attitude (in terms of cooperation and withdrawal from responsibility).

Thus, health becomes a reference point for creating the “subjective Self” and composes a biography of a human being who participates in social life by creating it, enabling the realisation of life plans (including intellectual–cognitive aspirations, moral and ethical references, and acts of love that exceed biopsychological conditions). Further, perceived health is constructively linked with experiencing the meaning of life (health, in a theological dimension, gives the possibility to read the complete meaning of life with respect to personal realisation in the relation both to oneself and to the human community). People live “for themselves, for their humanity—thus, strives to obtain everything for themselves, also to multiply their health in order to fully participate in their own project as persons; and does not strive for health in order to later sacrifice it or themselves, including own life, to realise a utopia of some impersonal being” (Pawłucki, 2002, p. 16).

Fourthly, health and forgiveness may be analysed in terms of communication. Since forgiveness is not an abstract act (the same applies to, e.g. healthy behaviour) and occurs in various contexts, one should consider the motivational constructs specific to these contexts. The first motivational construct, referring to the mutual interest in inner issues (e.g. the relationship: Self–Other), concerns treating one’s own personal state and situation as a way to understand the inner life of others. In a cognitive sense, it is possible to emphasise here the attempts to find a way to establish relationships through self-reflection. The second construct is based on the assumption that the decision to forgive can bring both anxiety and relief. Forgiveness will not solve all the problems, but it can reduce the cognitive tensions that accompany them. When a problem feels unresolvable, the situation related to it reduces one’s ability to think flexibly and leads to a state of cognitive exhaustion (Sedek & Kofta, 1990; Sedek et al., 1993). The loss of mental energy amplifies the seriousness of the problems one face and, by forgiving, a person can partially identify the distressing problem. Any decision depends greatly on how the person perceives their surroundings. Cognitive studies indicate that people usually perceive events not only as they appear to them but also through the prism of their own needs and biases (Barrett, 2004; Tremlin, 2006). Moreover, people have a natural tendency to form patterns of intentionality based on their experience with ambiguous stimuli (Epley et al., 2008). However, is forgiveness one of the important factors that enable the strengthening of health resources?

To conclude, health is—firstly—connected with seeking balance in the face of the burdens imposed on the organism by the surroundings (Heszen-Niejodek & Życińska, 2001); and secondly associated with using biological, psychological and social potentials to meet the internal and external requirements and achieve individual and social goals (Sęk, 1997).

### Health from a Biblical Perspective

Fifthly, spiritual health refers to faith as a fundamental value in human life. It is through faith that a person’s inner transformation occurs. This must affect the whole of their moral attitudes and behaviours (among others, those conducive to spiritual health) to the extent that one can state that faith, focal to human life, becomes a tremendous process of the person’s moral shaping. Faith not only leads to one’s transformation and emphasises spiritual life, but, in its deepest essence, it also creates this transformation. Hence, true faith cannot be in any way isolated from the ethos of individuals, understood as the totality of their moral actions, attitudes and behaviours, as well as their accepted hierarchy of values and the perception of themselves as persons. Experience and deeds are marked, animated and motivated by faith—the foundation of spiritual health. From this perspective, St Thomas Aquinas’ words are especially important; he defines health as a habitual disposition of the body, adequately related to the soul as its essence (Wróbel, 1999).

The aforementioned understanding of the notion of health (multidimensional) corresponds with its description found in the pages of the Holy Bible (Wróbel, 1999). In the Bible, three terms are used to denote health, namely “shālōm”, “marpě” and “rifộ”, although only the first of these is the proper equivalent of health (LXX: “eirệnē”; Wulgata: “sanitas”). The other terms “marpě” and “rifột” are rather used to refer to recovery or cure. In the Bible, one may also encounter the word “ischýō”—an equivalent to the notion of health, which, interpreted in the proper sense, means “to become strong”, while in the physical sense “to be strong”, and is used both for things (e.g. Is. 28:22) and persons (e.g. Judg. 1:28). On the other hand, the related word “ischyrós” means “strong” in reference to the following: God (e.g. Deut. 10:17), persons (e.g. Num. 13:18), animals (e.g. Pr. 30:30) and things (e.g. Is. 8:7). Simultaneously, in contrast to the ill, who have no life force, the healthy are figuratively called “ischýontes” (e.g. Mk. 2:17: “It is not the healthy who need the doctor, but the sick.”).

Interestingly, the word “shālōm” is most frequently translated as “peace”. It should be understood not so much in the sense of the classical Greek “eirệnē” (absence of war) as from the perspective of a situation opposite to the absence of the stability that guarantees prosperity, well-being and the sense of security. Hence, “shālōm” also signifies a state associated with good physical health (e.g. Ps. 38:3: “the sin has left no health in my bones”; Is. 57:18 “I shall heal him, I shall lead him, fill him with consolation, him and those who mourn for him”). Simultaneously, in other contexts, “shālōm” also broadly denotes a peaceful existence (e.g. Judg. 19:20), well-being, inner peace, welfare, prosperity and happiness (e.g. Ps. 73:3), which emphasise the dimensions of mental and social health. Although they represent two realities, peace and health (as “shālōm”) are nearly parallel notions because they have common features and are occasionally used interchangeably (e.g. Ps. 38:4; Jer. 6:14; 1 Sm. 1:17, 20:42; 2 Sm. 15:9; Mk. 5:34; Lk. 7:50, etc.). A notable example to this may be the phrase referring to the greeting “shālōm lekā” (meaning “peace be with you” and “be in health”), or the expression “lēk leshālōm” (meaning “go in peace” and “go in health”).

Equally worth noting are the words highlighting that health is more valuable than all riches, expressed in the canticle of praise of Sirach (30:14–16): “Better be poor if healthy and fit than rich if tormented in body. Health and strength are better than any gold, a robust body than untold wealth. No riches can outweigh bodily health, no enjoyment surpass a cheerful heart”. In the Bible, there are also other references to health, such as the fruit of moderation; submission to good advice, peace of heart, prudence and wisdom (e.g. Pr. 4:21–22: “Do not let them [good advice and prudence] out of your sight, keep them deep in your heart. For they are life to those who find them and health to all humanity.”); and references to wisdom (e.g. Pr. 3:7–8 “Do not congratulate yourself on your own wisdom, fear Yahweh and turn your back on evil: health-giving, this, to your body, relief to your bones.”).

### The Dilemma of Forgiveness and the Issue of Health

To understand the phenomenon of forgiveness, it is first necessary to address the phenomenon of harm, whose examples include betrayal, fraud, slander and theft. When analysing the consequences of the perpetrator’s decision, one can distinguish different types of consequences. Firstly, the victim may lose some goods, such as money or their good name. Secondly, the harm affects the victim’s dignity. The wrongdoer treats the victim as someone worse who does not deserve respect (Murphy, 2005; Murphy & Hampton, 2002). Thirdly, assuming that much harm is experienced within relationships that are important to the person, harm leads to the break down or weakening of these relationships, or simply reveals their weaknesses. Furthermore, one should note of the consequences relating to the victim’s psychological and spiritual spheres. Thus, fourthly, negative feelings, such as resentment, anger and even hatred, are evoked, prompting actions that the victim would not normally take (Griswold, 2014; Kolnai, 1974; MacLachlan, 2010). Fifthly, the victim has to face the decision to adopt a certain attitude towards the wrongdoer, the consequences of which are borne not only by the perpetrator and victim but also by third parties.

Just as harm has an aspect both external and internal to the victim, any reference to it also involves the external and internal spheres of the victim. On the one hand, the victims have to address the loss, the way they have been mistreated by the wrongdoers, and the future form of their relationship (which involves other persons too); and, on the other hand, they have to bring order to the sphere of their own feelings and solve the dilemmas that arise from them. One of the possible ways to address the harm is through forgiveness. Defining it is however difficult. Jeffrie Murphy—referring to the sermons of Joseph Butler (1827), which are the starting point for contemporary discussions on forgiveness—claims that forgiveness “involves the overcoming of anger and resentment”, that is, it takes place within a person. He goes on to distinguish mercy from forgiveness, which “involves the withholding of harsh treatment that one has the right to inflict” (Murphy, 1998, p. 697). In this notion, forgiveness is rather linked with the transformation of negative emotions evoked by experiencing harm. However, the philosophical discussion on this topic is not conclusive (Verbin, 2010). In many approaches, the focus is shifted to the victim’s attitude towards the perpetrator, especially since it is practically impossible to eliminate negative feelings linked with painful experiences, even among those who adopt a kind attitude towards their wrongdoers and seek to support them through actions.

Without going into a detailed discussion on the essence of forgiveness, one should note that it reveals several issues that the victim faces. Resolving these will be important not only for the person’s well-being, the state of their emotions or the shape of reality in which they will function in the future (Griswold, 2014). Referring to stereotypes, one can assume that seeking revenge will lead to a definitive break up in the relationship with the wrongdoer, while the decision to forgive will help rebuild a relationship that has been weakened or destroyed by the harm. Nevertheless, offering forgiveness may not also have this effect—it can send a message to the perpetrator that the mistreatment has been accepted by the victim (Murphy, 1998). The focal interest of this paper is to relate this decision to the victim’s health condition. It is also worth specifying several problems that have side effects on the individual’s health.

Firstly, health is linked to the sense of dignity and self-worth. The decision taken by the wrongdoer deprives the victim of some goods; more importantly, it conveys the message about the victim’s dignity and self-worth. A radical example of such a message was how prisoners in the Nazi concentration camps were treated. Taking their lives were done based on an ideology according to which they had neither dignity nor the right to live (Arendt, 2006). Actions that harm other people—even though they do not question their right to live—also refer to their dignity and self-worth. Infidelity in marriage implies that the spouse is not sufficiently important for the perpetrator to refrain from extramarital intercourse. Theft, on the other hand, not only leads to the loss of assets but also sends across a message that the victim is not valued enough to have their ownership respected. If health is conditioned by the sense of dignity and self-worth, then for the sake of health, the victim should restore the right hierarchy in the relationship between themselves and the wrongdoer. Given that, many authors criticise the understanding of forgiveness as a morally good and desirable act. They defend the value of resentment (Butler, 1827) and the drive to get even (Kekes, 2009; Mason, 2003; Murphy, 2003, 2005).

Secondly, health is conditioned by right relationships with significant persons, such as parents, siblings, spouses and children. Nevertheless, much harm (in fact, the most painful) is caused by the decisions of precisely these people. The weakening or ending of relationships with these persons due to unforgiveness can be a source of further suffering for the victim. While avoiding contact with a stranger, and even with a former friend, is possible and does not need to be a source of suffering (and it doesn't have to affect one’s health), breaking contact with loved ones and missing them can have negative health consequences.

Referring to the two above-mentioned considerations on deciding whether to forgive or not, it is not difficult to recognise that protecting one’s own dignity may contradict the wish to rebuild the relationship with the wrongdoer. Consequently, any resolution to the dilemma may lead to negative consequences for the victim’s health, whatever their source.

The third circumstance to be considered in addressing the relationship between forgiveness and health is the victim’s concern for others who will experience the consequences of forgiveness or the lack of it. Health, as indicated previously, is also related to the awareness that the well-being of others is influenced by the decisions taken by the person. A person’s health can be negatively affected by the knowledge that their decision has caused suffering to others, especially their closest ones. Harm and forgiveness are usually analysed in the context of a relationship between two persons directly involved in an event; however, they de facto occur in a broader social context. For instance, spousal betrayal affects not only the partner but also the children born of the relationship and other persons related to the spouses, such as the parents of the harmed and the perpetrator. Nevertheless, not only the wrongdoer’s act is significant for these persons’ well-being. The victim, in deciding whether to forgive, also adopts an attitude significant for their closed one’s well-being. Keeping the unfaithful spouses away from the children, portraying them in a negative light, making contacts with them impossible and preventing their participation in events important for the children can also be a source of suffering for children for whom the unfaithful spouse remains the only father or mother. When deciding on the attitude to adopt towards the unfaithful spouse, the betrayed person experiences dilemmas involving the consequences of their decision for the children’s good.

Given this circumstance of deciding whether or not to forgive, it can be noticed that the decision combined with the victim’s concern for their own dignity and self-worth can also be a source of the dilemma regarding the best solution to follow in a given situation. Whether the victim seeks to get even with the wrongdoer or forgive, particular resolutions can lead to the negative self-evaluation of their impact on the third parties’ well-being, or on concern for their own dignity, thus negatively influencing the victim’s health.

For the victim’s dignity to be protected in the context of experiencing harm and deciding to forgive for the sake of the relationship with the wrongdoer or for others’ good, it has been frequently emphasised that the decision to forgive should be made after the perpetrator has acknowledged the guilt, expressed a readiness to apologise, changed their attitude and sought reparation (Barnes, 2002; Griswold, 2014; Kolnai, 1974). The fulfilment of even one of these conditions could suggest that the perpetrator has recognised the victim’s dignity and worth and taken a proper attitude to the victim in the relationship. Consequently, the decision to forgive would not imply a threat to the victim’s dignity and would in fact secure their proper value in the relationship with the perpetrator. However, in reality, this conditioning of the decision to forgive can cause additional difficulties for the victim. When the wrongdoer does not show remorse, change their own attitude and doesn’t seek to repair the harm done, the victim becomes trapped in an attitude of unforgiveness (Wolfendale, 2005). These problems trigger a discussion between the proponents of conditional and unconditional forgiveness (Garrard & McNaughton, 2003; Hieronymi, 2001; Holmgren, 1993).

### The problem with Feelings Against Forgiveness and the Issue of Health

The fourth circumstance that needs to be emphasised in the reflection on the relationship between harm, forgiveness and health is the very nature of forgiveness. The act is expressed through the attitude towards the wrongdoer but also encompasses the sphere of human feelings. From the perspective of human health, individuals must achieve inner harmony between their feelings and what is regarded as the best solution in a given situation. The dissonance between feelings and reason can have negative consequences for health. Harm evokes negative emotions that can make the victim wish to get even. Consequently, the wronged persons are in a specific situation in which, to be able to forgive (if this is considered the best solution, e.g. for the sake of third parties), they must face negative emotions prompting to take the opposite decision (Szigeti, 2014; Warmke, 2015). The inability to forgive—if forgiveness is perceived by reason as desirable in a given situation—due to the negative emotions evoked by harm can cause tension, thereby implying negative consequences for the victim’s health.

Although this analysis focuses on the potential dilemmas experienced by the subject and the tension between the decision based on reason and the action triggered by feelings, when concluding the analysis, it is worth noting that forgiveness in itself may be a source of health for the individual making such a decision. This is a topic that requires a separate study, so we do not want to discuss it broadly, but it is worth noting that the situation of experiencing harm—due to the negative feelings evoked in the victim by the perpetrator—implies the victim’s loss of harmony, order in life and, in a sense, control over relationships. Conversely, forgiveness can be understood as regaining control over the relationships between the victim and the perpetrator. For the victim who decides to forgive, this can be a condition for achieving a state equated with health. For that to happen, the decision to forgive must be mature.

To summarise, the issue of the relationship between the victim’s health and the decision to forgive encompasses twofold problems. The first problem comprises identifying the attitude best for the victim from their own perspective and that of a possible relationship with the wrongdoer and third parties experiencing the consequences of the decision. This means determining the attitude that will promote the victim’s personal development, as well as social relationships. The second problem deals with identifying the point of reference for this decision. Such a benchmark would be a source of strength when dealing with the feelings prompting the opposite action. Religion fits into the context of decision-making about the attitude towards the wrongdoer; it indicates a specific way of interpreting reality and creates a new network of references, motivating particular attitudes.

### Christianity and the Dilemma of Forgiveness

In Biblical texts, as the primary source of Christian doctrine, forgiveness is regarded positively. Moreover, Christianity has formulated the imperative to forgive. Therefore, it is perceived as a religion that has ascribed a new meaning to forgiveness and that promotes this decision (Bash, 2007; Nussbaum, 2016). However, in the context of the aforementioned dilemmas related to forgiveness, it seems justified to doubt the positive value of the impact that the decision to forgive may have on the victim’s well-being and health. This section attempts to confront these doubts.

The best-known passages in the Bible dedicated to forgiveness can be interpreted as a call to take such a decision, and to do so unconditionally; they even present forgiveness as a condition for obtaining absolution for one’s own sins. Jesus, asked by Peter: “Lord, how often must I forgive my brother if he wrongs me? As often as seven times?”, responds: “Not seven, I tell you, but seventy-seven times.” (Mt. 18:21–22). In turn, when explaining the words of the Lord’s Prayer, Jesus says: “If you forgive others their failings, your heavenly Father will forgive you yours; but if you do not forgive others, your Father will not forgive your failings either.” (Mt. 6:14–15). This extract corresponds well with other passages. The book of Kohelet records the following wisdom: “Pardon your neighbour any wrongs done to you, and when you pray, your sins will be forgiven. If anyone nurses anger against another, can one then demand compassion from the Lord?” (Ecc. 28:2–3), and Matthew’s story of the unforgiving servant ends with a strong conclusion: “And that is how my heavenly Father will deal with you unless you each forgive your brother from your heart” (Mt. 18:35). Furthermore, the message about forgiveness is reinforced by the Biblical description of the pattern of forgiveness. When explaining why he did not take up the mission assigned to him by God, Jonah (4:2) states: “I knew you were a tender, compassionate God, slow to anger, rich in faithful love”. This image of God is visible in the New Testament, in which one finds descriptions of people granted forgiveness by Jesus (Bash, 2007; Ely, 2004; Escher, 2013).

The verses quoted here, torn from the Biblical message as a whole, may suggest the necessity of making a decision that disregards either the victims’ welfare (exposing them to further harm) or their feelings, whose intensity may impede or prevent the decision to forgive. Thus, it is easy to assume that the decision taken against intense feelings and implying the possibility of new harm (i.e. putting the forgiver in a situation of threat to their well-being) may in many cases negatively affect the victim’s health. Consequently, such a decision may seem irrational and naive. This decision conditioned by the victim’s faith is sometimes justified by the hope that the wrongdoer’s attitude will change under the influence of the good experience from the victim (Giannini, 2017); yet, this hope can also be irrational (Starnawski, 2009), and the decision to forgive imprudent (Horowski, 2019). If, therefore, the Christian understanding of forgiveness would refer only to the above-mentioned words, then one could conclude that Christianity, by calling for forgiveness, neither supports the individual’s development nor contributes to the concern for their health, but it forces them to face the dilemma of rationality and faith, causing unnecessary psychological stress.

However, looking at other Biblical excerpts, one can notice that wrongdoing is not unconditionally forgiven. Firstly, descriptions of the evil done contain pictures of the punishment experienced by the wrongdoer. Cain, who kills his brother Abel, becomes “a restless wanderer … on the earth” (Gen. 4:12). David, having sinned with Bathsheba and murdered her husband, loses a child (Sam. 12:14). Secondly, forgiveness is only given when the perpetrator has understood the evil done and changed their attitude. The prodigal son receives his father’s forgiveness only after experiencing the humiliation of breeding pigs and eating what they eat (Lk. 15:16–17); the blatant sinner obtains forgiveness from Jesus after anointing his feet with oil (Lk 7:36–50); and the thief hanging on the cross next to Jesus hears the words, “Today you will be with me in paradise”, only after confessing that the punishment he is suffering is just (Lk. 23:39–43).

Thirdly, the Bible contains descriptions of a decisive response to evil and an encouragement to rebuke the wrongdoer. To illustrate this, one can recall the destruction of Sodom and Gomorrah (Gen. 19:28), driving the merchants out of the temple (Jn. 2:15), or the words on fraternal correction, which take into account the possibility of severing the relationship with the wrongdoer: “If your brother does something wrong, go and have it out with him alone, between your two selves. If he listens to you, you have won back your brother. If he does not listen, take one or two others along with you: whatever the misdemeanour, the evidence of two or three witnesses is required to sustain the charge. But if he refuses to listen to these, report it to the community; and if he refuses to listen to the community, treat him like a gentile or a tax collector” (Mt. 18:15–17). These formulations, in turn, encourage a decisive attitude towards evil, which, if adopted towards a stranger, may not cause the victim much trouble, but it may involve numerous dilemmas and inner conflicts if the wrongdoer is close to the victim. Consequently, adopting this attitude towards a close person can negatively affect the victim’s health.

Contemporary studies on Biblical forgiveness express the difficulties with formulating a clear concept of the reality of forgiveness (Couenhoven, 2010), which were described in the previous section. For example, they ask whether the essence of forgiveness lies in the change of attitude towards the wrongdoer, or whether a change of feeling towards the perpetrator is necessary for forgiveness. Behind this dilemma lies another question concerning whether forgiveness is similar to cancelling a debt (which does not require a change of feelings towards the debtor), or whether it is linked to restoring relations between the perpetrator and the victim (which already involves changes in feelings). The Biblical account offers two above-mentioned meanings of the term forgiveness (Louw, 1997). Forgiveness as the cancellation of a debt was practised especially in the Yom Kippur ritual (Pilarczyk, 2016), but the forgiveness based on restoring the relationship between the victim and the wrongdoer was also of interest to the Israelites, as testified to by the story of reconciliation between Joseph and his brothers, who sold him into slavery (Gen. 37:28). David Konstan (2010), analysing how the idea of forgiveness has developed, states that the contemporary idea of transgressive forgiveness, which highlights the change in feelings towards the perpetrator, was unknown in antiquity (among both the Greeks, the Romans, and the Hebrew world). However, when looking at the stories on harm and forgiveness regarding Biblical characters, one can easily notice that their inner transformation involved an emotional component.

Regardless of which of the meanings of forgiveness is used earlier or more popular in the Bible, a deeper reflection on the stories of forgiveness allows one to formulate several conclusions about the attitude that should be adopted towards the wrongdoer, which can help deal with the dilemmas described in the previous section; dilemmas whose resolution can negatively affect human health.

Firstly, the decision to forgive should always be taken for improving the wrongdoers’ well-being. However, this does not always mean impunity. The Bible understands individuals’ well-being as building morally coherent relationships with God and other people. Harm done to another person violates well-being of not only the victim but also that of the wrongdoers, among others, by destroying their relationships. If the perpetrators do not understand what is best for them, and that by hurting others they hurt themselves, actions must be taken so that they discover the right moral order. In some cases, this may mean inflicting severe punishments for the deed; in others, it may mean keeping distance from the wrongdoers; and in some others, if the right moral order has been discovered and obeyed by the wrongdoers, this may lead to the decision to forgive them unconditionally. It is worth noting that the Bible does not exclude punishment; only revenge is condemned. The very first pages of the Bible instruct how to treat the wrongdoer. Adam and Eve (expelled from Paradise) receive garments of skins prepared by God (Gen. 3:21), while Cain (condemned to wander) receives a mark that is intended to prevent any individual from killing him (Gen. 4:14–15).

Secondly, forgiveness is not equated with reconciliation, even though these are close decisions. If forgiveness comprises concern for the wrongdoer’s good and is in a sense an effort to help the offender develop the right attitudes towards other people, and concern for that good requires keeping distance from the perpetrator, then in some cases, forgiveness may not be linked with reconciliation. Moreover, the victim may forgive, that is, adopt an attitude of concern for the wrongdoer’s well-being while not communicating this to the perpetrator. In such a case, the victim uses the wrongdoer’s lack of awareness of forgiveness as a means to their well-being. Returning to the situation that occurred when Jesus was hanged on the cross, one could ask if the scoundrel who felt no remorse and mocked Jesus (Lk 23:39) did not experience forgiveness on his part, or perhaps forgiveness did occur, but the perpetrator had not matured to hear it.

Defining forgiveness as adopting a specific attitude towards the wrongdoer (an expression of concern for that individual) and, consequently, separating this attitude from both communicating forgiveness to the wrongdoer and from reconciling with them, may solve most dilemmas relevant to the victim’s health, as analysed in the previous section. Firstly, the victim may forgive but not communicate their decision to the wrongdoer if they are convinced that such a communication would endanger either their own well-being or that of others affected by the decision. Moreover, in some circumstances, the absence of such communication may express concern for the wrongdoer. Secondly, the victim can forgive and wish to rebuild the relationship with the wrongdoer but keep a distance from them due to the perpetrator’s visible immaturity in the relationship. Thirdly, the victim who keeps their distance may act for the perpetrator’s benefit so as to indirectly support other people affected by this situation; for example, children born of a broken marriage and experiencing the effects of how one of the parents copes with life’s problems better or worse. Furthermore, it is worth noting that separating forgiveness from communicating forgiveness and therefore from reconciliation, also resolves the issue of prerequisites for forgiveness. The victim is not constrained by these conditions in their decision. The person can forgive and regard these conditions (perceived as a premise for forgiveness) as necessary for communicating the decision to forgive or the desire to reconcile.

### Christianity and the Problem with Feelings Against to Forgiveness

The last dilemma concerns the conflict between the feelings and the decisions supported by reason on the attitude to adopt towards the wrongdoer. These emotions (sometimes very intense) can prevent one from taking actions supported by reason. To deal with this inner conflict, the victim needs not so much an intellectual solution as a factor supporting the desire to “convince” feelings (the verb “convince” is understood here in an analogous sense). The greatest difficulties in dealing with feelings may arise in those cases where the wrongdoer is a stranger, a person with whom the victim does not wish to maintain a relationship. Forgiveness in a relationship with someone close may be motivated by a longing for that person. When it comes to strangers, the problem is more complex. One of the proposed justifications for forgiveness towards strangers is interpersonal solidarity (Garrard & McNaughton, 2003). In Christianity, the factor that supports the act of forgiving strangers is God, understood as a person and as one of the poles in the relationship formed by the victim (Ratzinger, 2007). To understand the influence of this relationship on the forgiveness process, one can use the example of interpersonal relationships. A specific relationship is that of a man with the child of his beloved woman. On the one hand, he does not have the bond with the child like a father has, but, on the other hand, he cannot be indifferent towards the child because of the person he loves. When he is hurt by the child, who frequently perceives him as an intruder at home, the relationship with the child’s mother can motivate forgiveness. Human relationships are similarly understood when viewed from a religious perspective. Although the wrongdoer can remain a stranger to the victim, it is impossible to ignore the awareness that the wrongdoer is loved by God just as much as the victim is. The decision to forgive is therefore not taken due to the relationship with the wrongdoer but due to the love for God. Returning to the theme of the victim’s health, one can say that the relationship with God (love for God) may be an essential factor motivating the victim to adopt a positive attitude towards the wrongdoer and may thus weaken the negative tension between the feelings evoked by the harm and the decision to forgive supported by reason as prudent.

To conclude this analysis, it is worth noting that the decision to forgive involves both cognitive and desire-related powers. Thinking about human health as conditioned by the right decisions, attention should be paid to both these powers. Looking at the wrongdoer through the prism of love God surrounds them with and adopting attitudes that are ill-considered and naive can negatively affect the victim’s health. On the other hand, a religiously conditioned decision to forgive taken by a person characterised by traditional religiosity (one not based on a relationship with God) may create greater tensions related to the intensity of feelings that in turn lead them to act against forgiveness, besides negatively affect the person’s health.

## Summary

To summarise the above analysis, several threads must be emphasised. Forgiveness is one of the ways to understand and practise religion. However, one cannot question either the key role it plays or the complexity of its theological and psycho-social determinants. Forgiveness reflects the transformations that the person’s perception of the world undergoes in the course of their personal (including spiritual) development. Whether in childhood, adolescence, adulthood or old age, forgiveness plays an important role in the person’s struggle with life’s problems. It reflects the acts as well as the cognitive content and motivations of people who forgive.

If health is understood holistically, as conditioned not only by physical factors but also by psychological and spiritual ones, dealing with the decision on whether to forgive or not is an important determinant of a given person’s health. Linking forgiveness with its positive impact on human health may be considered naive and unwise since forgiveness may evoke in an immature forgiver numerous dilemmas and tensions that negatively affect their health. Christianity, which not only promotes forgiveness but also turns it into a moral requirement, is also part of the context in which many decisions about forgiveness are made. In this way, Christianity can reinforce the dilemmas and inner tensions faced by victims, which is crucial for their health.

The conducted analysis draws the conclusion that the Bible offers a concept of forgiveness by which the above-mentioned dilemmas can be solved. In this concept, forgiveness is distinguished from informing the wrongdoer of the act and separated from reconciliation. At the same time, Christianity based on the relationship with God can help the victim find the strength to overcome the tension between the decision to forgive and the emotions caused by the harm. This paper can thus confirm the thesis that Christianity has the potential to support the health of a person who struggles with the experience of harm and who seeks to resolve the problems arising from this experience. Not every religiously conditioned decision to forgive is, however, prudent and actually benefits the victim’s health.

Among all possible individual religious expressions, forgiveness has the actual dimension of an act full of behavioural expression. It is a rich and complex subject of research. The authors hope, therefore, that these reflections on human health in the context of the forgiveness dilemma will inspire further concepts and encourage studies that will help better understand and improve people’s relationships with others, and individuals’ connection with health in particular (e.g. patterns of health-promoting behaviour).
